# Impact of Tobacco Smoking on Outcomes of Radiotherapy: A Narrative Review

**DOI:** 10.3390/curroncol29040186

**Published:** 2022-03-24

**Authors:** Adrian Perdyan, Jacek Jassem

**Affiliations:** 1 International Research Agenda 3P Medicine Laboratory, Medical University of Gdańsk, 80-211 Gdańsk, Poland; perdyan.adrian@gumed.edu.pl; 2 Department of Oncology and Radiotherapy, Medical University of Gdańsk, 80-214 Gdańsk, Poland

**Keywords:** tobacco smoking, smoking cessation, radiotherapy, cancer, treatment outcome

## Abstract

The carcinogenic role of tobacco smoking is well recognized, but the detrimental effects of continued smoking after a cancer diagnosis have been underestimated. Radiotherapy is among the main treatment modalities for cancer. We reviewed the literature data concerning the impact of tobacco smoking on treatment outcomes in radiotherapy-managed patients with various malignancies. Most of the analyzed studies demonstrated the detrimental effect of smoking on overall survival, tumor control, quality of life, treatment toxicity, and the incidence of second primary malignancies. Healthcare professionals should use the cancer diagnosis and treatment as a teachable moment and recommend their patients to immediately cease smoking. Wherever possible, cancer patients should undergo an intensive smoking-cessation program, including behavioral and pharmacologic therapy.

## 1. Introduction

Tobacco smoking is a well-documented causative factor for at least 13 malignancies [1]. More than 60% of cancer patients are current or former smokers [2]. Numerous studies and meta-analyses have demonstrated that continuing smoking after a cancer diagnosis negatively impacts survival and quality of life (QoL), while increasing treatment toxicity and the risk of secondary malignancies [3,4,5]. Radiotherapy (RT) is one of the main treatment modalities in cancer, contributing to around 40% of cures [6]. Approximately half of cancer patients will receive RT during the course of their disease [7].

The purpose of this article is to review the impact of tobacco smoking on RT outcomes in various malignancies. The examples provided here should be considered illustrative, as no comprehensive analysis of the literature pertaining to the topic was attempted. 

## 2. Materials and Methods

The narrative review was done according to SANRA guidelines (https://researchintegrityjournal.biomedcentral.com/articles/10.1186/s41073-019-0064-8; accessed 15 September 2021). In October 2021, we performed a search using PubMed, Scopus, and Google Scholar. We used following search query: “radiotherapy AND (tobacco OR cigarette) AND smoking AND cancer”. Additionally, articles were found in references of included articles and by Google search using “smoking during radiotherapy” during following months. Table 1 presents the summary of included studies.

We included studies reporting (1) the number of active smokers during RT or chemoradiotherapy (CHRT) and non-smokers or past smokers as a control group, and (2) the outcomes for both group with regard to at least one of the specific endpoints: overall survival (OS) (Table 2), progression-free-survival (PFS), distant-free-survival (DFS), locoregional control (LRC), distant control (Table 3), risk of secondary primary cancer (SPC) (Table 4), and treatment toxicity and complications (Table 5).

Articles were excluded if authors did not report any of the data mentioned in the including criteria. If multiple publications of the same cohort were available, only the latest publication was selected. Studies in languages other than English were excluded from this narrative review. 

## 3. Lung Cancer

Of all malignancies, lung cancer is the most greatly associated with tobacco smoking. This association is stronger for squamous and small cell lung cancer (SCLC) than for adenocarcinoma and large cell carcinoma [65]. 

### 3.1. Non-Small Cell Lung Cancer

The impact of tobacco smoking on OS in non-small cell lung cancer (NSCLC) patients managed with RT or CHRT is unclear. In a study by Tsao et al., including 1370 advanced NSCLC patients (497 of whom were administered CHRT), the median OS in never, former, and current smokers in the CHRT cohort was similar (1.5, 1.5, and 1.4 years, respectively) [8]. There were also no significant differences in response rates between these groups (63%, 59%, and 50%, respectively). In a study by Nguyen et al., including stage II/III NSCLC patients administered postoperative RT, smokers, compared to non-smokers, had worse five-year local control (LC): 70% versus 90%, respectively (*p* = 0.001 and LRC: 52% versus 77% (*p* = 0.0006) [9]. In a study including stage I patients treated with stereotactic body radiation therapy (SBRT), no statistical differences were observed in terms of three-year LC rate (52% versus 56%) and OS between patients with a history of smoking and never smokers [10]. However, SBRT-induced toxicity occurred in 14.5% of current or past smokers and none of the never smokers. In another study including NSCLC patients managed with SBRT, smoking cessation was associated with improved two-year OS compared to continued smoking (78% versus 55%, respectively; *p* = 0.014) [11]. Finally, in the study by Miller et al., smoking status was not associated with distant failure risk [12].

Fox et al. showed inferior OS in smoking compared to non-smoking stage I/II NSCLC patients managed with RT or CHRT (41% versus 56%, respectively; *p* = 0.01) [13]. In a recent Russian study of Sheikh et al., the adjusted median OS and five-year OS in smokers and quitters were 4.8 years versus 6.6 years (*p* = 0.001) and 49% versus 61%, respectively (*p* = 0.001), and the five-year PFS was 44% versus 54%, respectively (*p* = 0.004) [14].

In a study by Rades et al., smoking during RT was associated with worse two-year LCR (34% for smokers versus 59% for non-smokers; *p* < 0.001), but this was not translated into a significant OS difference [15]. Paradoxically, a few studies reported higher rates of radiation-induced pulmonary (RP) toxicity in non-smokers than in smokers [16,17,18]. These observations were contrary to the Monson et al. study, which showed a 23% and 0% prevalence of RP toxicity in smoking and non-smoking patients, respectively (*p* < 0.01) [19]. In a study by Sarihan et al., the prevalence of infections was highly associated with tobacco exposure (*p* = 0.001) [20]. In turn, frequent infections were associated with inferior OS (median 9 versus 13 months, respectively; *p* = 0.042).

Kawaguchi et al. reported more than five-fold higher relative risk (RR) of SPC in stage III NSCLC smokers treated with CHRT compared to the general population [21]. The risk in those who quit smoking was not significantly higher than the risk in the general population. 

### 3.2. Small Cell Lung Cancer

The first report on the impact of smoking during CHRT or chemotherapy (CHT) in SCLC was published by Johnston et al. in 1980 [22]. The median OS for smokers, quitters at diagnosis, and quitters before the diagnosis, was 47, 52, and 70 weeks, respectively (*p* < 0.04). In the study of Videtic et al., including limited-disease SCLC patients managed with CHRT, the median OS in former and continuing smokers was 18.0 months and 13.6 months, respectively (*p* = 0.002), and five-year OS was 8.9% and 4.0%, respectively (*p* = 0.0017) [23]. Patients who continued smoking during treatment and experienced a treatment-related break had the poorest OS. In another study, the RR of second lung cancer in continued smokers and quitters was 9.1 and 21, respectively, compared with general population [24]. In a study by Kawahara et al., including SCLC patients managed with CHRT or CHT, SPC occurred in 33% of patients who continued smoking, in 10% of patients who quit smoking, and in none of the never-smokers [25]. The risk of developing SPC in patients who continued smoking was significantly higher than in non-smokers (*p* = 0.03). 

## 4. Head and Neck Cancer

Around 70% to 80% of head and neck cancers (HNCs) are linked to tobacco smoking [66]. In a study by Browman et al., HNC patients who continued to smoke tobacco during RT or CHRT had a lower complete response rate (45% versus 74%, respectively; *p* = 0.008) and two-year OS (39% versus 66%, respectively; *p* = 0.002) than those who abstained from smoking [26]. In a subsequent analysis of this study in another cohort of HNC patients, the median OS in abstainers and very light smokers was 42 months compared to 29 months in light, moderate, or heavy smokers (*p* = 0.07) [27]. However, smoking during RT was not an independent negative predictive factor, as opposed to smoking status at baseline. Somewhat surprisingly, in the study of Kawakita et al., including HNC patients managed with RT or CHRT, non-smokers had significantly worse OS than light smokers (*p* = 0.016), which raised doubts regarding the allocation of patient groups [28]. In a prospective study from Denmark, smokers had inferior OS and LRC compared to non-smokers (*p* = 0.03 and 0.02, respectively) [29]. 

In a study by Fortin et al., including HNC patients managed with definitive or postoperative CHRT, the OS was impaired in current and former smokers versus never-smokers (*p* = 0.000001) [30]. LC was lower in current compared to former smokers (*p* = 0.0001). Additionally, the LC curve of current smokers continued to decrease over time, contrary to the curve of former smokers, which reached a plateau after three years. For never, former, and current smokers, the five-year LC rates were 75%, 80%, and 67%, respectively (*p* < 0.0000001). 

The detrimental impact of tobacco exposure was also reported in oropharyngeal cancer patients participating in phase III clinical trials of Radiation Therapy Oncology Group (RTOG) 9003 and 0129 [31]. Patients with an exposure higher than 10 PYs at diagnosis managed with definitive RT had an approximate doubled risk of death compared to those with up to 10 PYs (*p* = 0.001), corresponding to an absolute 30% difference in five-year OS. Smoking remained an important predictor of OS and PFS after adjustment for tumor p16 status and other significant prognostic factors (*p* < 0.001 and <0.001, respectively). In the RTOG 0129 study, tobacco exposure negatively impacted OS in patients administered CHRT (*p* < 0.05). In the study cohorts of RTOG 0129 and 9003 trials, the five-year locoregional failure rate was higher in patients smoking more than 10 PYs versus up to 10 PYs at diagnosis (29% and 12%, respectively; *p* = 0.001 and 48% versus 26%, respectively; *p* = 0.01). Additionally, smoking exposure at diagnosis was the only significant factor associated with a higher risk of developing an SPC. In a study including 1875 human papillomavirus-positive oropharyngeal cancer patients managed with CHRT or RT, current smokers had inferior OS, PFS and DFS than non-smokers [32]. Finally, in a meta-analysis comprising 24 studies and 6332 HNC patients, smoking during or after RT was associated with a nearly doubled risk of death (*p* < 0.0001) and more than doubled risk of locoregional failure (*p* = 0.0005) [33]. 

Chen et al. showed superior three-year PFS in HNC patients managed with definitive CHRT who quit smoking after diagnosis, compared to continuing smokers (61% versus 34%, respectively; *p* = 0.05) [34]. Continuing smokers also had more grade ≥3 acute toxicities (*p* = 0.01) and a higher probability of gastrostomy or tracheostomy (*p* = 0.03). In a study by Egestad et al., including HNC patients administered postoperative RT or CHRT, smoking status was associated with increased fatigue, pain, speech disturbances, mouth opening problems, and poorer cognitive function [35]. Likewise, in a Danish study, smokers, compared to non-smokers, showed worse cognitive function, nausea/vomiting, dyspnea, appetite loss, diarrhea, and weight loss (*p* < 0.05) [36]. Finally, in a study by Silveira et al., continued smoking decreased patients’ QoL by negatively affecting burden (*p* = 0.003), mental health (*p* = 0.03), and fatigue (*p* = 0.028) [37]. In a study including 10,381 nasopharyngeal cancer patients administered intensity-modulated RT or CHRT, current smokers had higher risk of death and recurrence compared to never-smokers (*p* = 0.003 and 0.027, respectively), whereas the risk of metastasis was higher in the former and current smokers (*p* = 0.031 and 0.019, respectively) than in never-smokers [39]. 

In the study by Al-Mamgani et al. in T1a glottis cancer patients, continued smoking smokers, compared to posttreatment quitters, had worse LC (81% versus 94; *p* = 0.001, OS (36% versus 70%; *p* < 0.001) and increased incidence of SPC (21% versus 12%; *p* = 0.003) [40]. Additionally, quitters showed better voice quality during two-year follow-up (*p* < 0.001 for all time points). In another study including patients with T1 glottis cancer, tobacco smoking was the single most important factor influencing the rate of RT-related complications (*p* = 0.031 after Bonferroni correction) [41]. The adjusted ten-year complication rates were 28% for continuing smokers, 11% for those who quit after RT, 14% for those who quit before RT, and 26% for never smokers. In a study by Porock et al., smoking HNC patients were more likely to develop oral mucositis (*p* = 0.03), and its severity was associated with tobacco exposure (*p* = 0.008) [42]. In a study by Rugg et al., mucositis lasted longest in patients who smoked during RT (23.4 weeks in current smokers, 18.3 weeks in those who temporarily abstained, 13.6 weeks in those who stopped before RT, and 12.4 weeks in never smokers) [43]. In a recent meta-analysis, HNC patients who ceased smoking developed less late toxicities, particularly osteoradionecrosis, than those who continued smoking [33]. In another study including HNC patients treated with RT, surgery, or a combination, current smokers had a significantly higher risk of death and SPC than never or former smokers [44]. 

## 5. Breast Cancer

In a cancer registry–based study including 10,676 breast cancer (BC) patients administered postoperative RT, the mortality was significantly higher for current or former-smokers than in never-smokers [45]. Additionally, smoking increased the risk of RT-induced SPC. In a study including 1029 BC patients subjected to lumpectomy and RT, those who continued smoking at the time of RT had a 20% 15-year risk of SPC compared to 16% for those who were non-smokers (*p* = 0.07) [46]. The 15-year risk of developing lung cancer after RT was 0.26% for non-smokers, 4.7% for former smokers, and 6.0% for smokers at the time of RT (*p* = 0.06). In a study by Sharp et al., including BC patients receiving postoperative RT, tobacco smoking was an independent factor associated with severe acute skin reactions (*p* = 0.031) [47]. Patients with such reactions showed a worse QoL (*p* = 0.007) and higher levels of pain and insomnia (*p* < 0.05). In another study, smoking and RT showed more than an additive effect on the risk of myocardial infarction (*p* = 0.039) [48]. In a meta-analysis including 71 studies using adjuvant RT, smoking was associated with worse post-reconstruction outcomes, such as higher failure rate of capsular contracture reconstruction, and other major complications. [49]. 

## 6. Prostate Cancer

Two studies demonstrated a negative effect of tobacco smoking in prostate cancer (PC) managed with brachytherapy [50,51]. The study by Taira et al. showed a more than doubled RR of death in former versus never-smokers (*p* = 0.007) and more than four-fold RR in current versus never-smokers (*p* < 0.001) [50]. In the second study, the HR for OS was 1.4 (*p* = 0.017) for never versus former smokers and 2.9 (*p* < 0.001) for never- versus current smokers [51]. In another study including patients managed with external RT, HR for OS was 1.72 (*p* = 0.08) and for distant control 5.24 (*p* = 0.003) in current versus never-smokers [52]. In a study including 61 PC patients managed with definitive external beam RT, six-year OS in non-smokers and smokers was 26% and 11%, respectively (*p* = 0.009) [53]. Alsadius et al. reported a higher prevalence of abdominal cramps (*p* = 0.004), defecation urgency (*p* < 0.001), sensations of bowel not completely emptied after defecation (*p* = 0.003), and sudden emptying of bowels into clothing without forewarning (*p* = 0.003) among smoking compared to non-smoking PC patients managed with external-beam RT [54]. In a study by Boorjian et al., the HR of secondary bladder cancer was 3.65 in smoking patients who received RT [55]. Finally, in the meta-analysis by Foerster et al., including 22,546 PC patients managed with prostatectomy or RT, the HR of biochemical recurrence in current and former-smokers receiving RT was 1.50 (1.20–1.88) and 1.10 (0.94–1.28), respectively [56]. 

## 7. Cervical Cancer

In a study by Mayadev et al., two-year pelvic control and DFS in heavy smoking cervical cancer women administered concurrent CHRT (>21 PY) were inferior to non-smokers (*p* = 0.004 and 0.011, respectively) [57]. In a study by Eifel et al., including International Federation of Gynecology and Obstetrics stage I and II patients managed with RT, heavy smoking was the strongest independent predictor of all complications (*p* < 0.0005) [58]. The most striking smoking-related adverse effect were small bowel complications (for smokers of ≥1 pack per day: *p* < 0.0005). In turn, in the study by Fyles et al., smoking status was not associated with PFS and LC [59]. 

## 8. Other Malignancies

In a large Chinese series, smoking esophageal cancer patients managed with CHRT had a five-year OS of 32% compared to 51% in never-smokers (*p* = 0.01) [60]. There was no significant difference in OS between former and current smokers. Heavy smokers (>47.5 PY) had a poorer five-year OS than light smokers (16% versus 38%, respectively; *p* < 0.001). In a study by Lerman et al. including anal cancer patients administered RT or CHRT, smoking was the only independent factor significantly related to PFS (*p* = 0.013) [61]. In a study by Leeuven et al., patients who smoked more than 10 PY after a diagnosis of Hodgkin lymphoma experienced a six-fold elevated risk of secondary lung cancer compared with patients who smoked less than one PY (*p* = 0.03; adjusted for RT dose) [62]. In a study by Peppone et al. including BC, genitourinary cancer, lung cancer, gastrointestinal cancer, and other malignancies managed with RT, CHT, or both, the mean total symptom burden during treatment was significantly greater in smoking than in non-smoking patients (46% versus 41%; *p* = 0.048) [63]. Patients who quit smoking before treatment had a total symptom burden comparable to non-smokers. In another study including BC, HNC, and anorectal cancers, current smokers had higher rates of RT-induced skin reactions than former smokers [64]. Smoking was associated with higher RTOG scores (*p* < 0.0001), erythema meter mean (*p* = 0.009), and mean diary score, including pain, itching, burning, and sleep disturbances (*p* = 0.016).

## 9. Discussion

Most of the presented studies demonstrated the adverse impact of smoking on OS, tumor control, QoL, treatment toxicity, and the incidence of SPC in cancer patients managed with RT. Detrimental effects of smoking concern definitive and postoperative (CH)RT in both smoking-related and unrelated malignancies. Early cessation after cancer diagnosis improves the efficacy of RT and QoL and decreases the risk of SPC and mortality from other tobacco-related diseases.

Somewhat surprisingly, as opposed to other malignancies, the detrimental effect of tobacco smoking on OS in NSCLC was inconsistent. This may be due to a very high prevalence of tobacco smoking in this group, making comparisons with much less numerous groups of non-smokers challenging. More uniform, both in lung cancer and other malignancies, was the adverse impact of continued smoking on the risk of SPC and treatment tolerance.

The harmful effect of smoking on treatment toxicity and induction of SPC is well recognized and applies to various malignancies and therapies. RT is a local treatment modality, and its beneficial effects are basically via locoregional tumor control. Many studies presented in this review indicate that smoking may adversely impact locoregional tumor control. The mechanisms underlying this effect remain uncertain. One of the proposed reasons is decreased tumor oxygenation, a critical factor for the efficacy of RT [67]. This effect may be caused by smoking-induced respiratory insufficiency before RT, exacerbated by increased carboxyhemoglobin levels in patients who continued smoking during RT [68]. 

We are aware of several limitations of our work. First, due to a large body and diversity of literature data, we have not attempted a comprehensive subject analysis. Nevertheless, we have done our best to objectively present studies that did and did not demonstrate a negative impact of tobacco smoking. Second, almost all presented studies are observational and retrospective, thus some potentially relevant data might have been missed. In the majority of studies, the self-reported smoking status was not validated biochemically. Some studies included mixed populations of patients who did and did not receive RT, and the subset analyses might have insufficient power to detect significant differences. In several studies the number of patients who quit or continued smoking during treatment, as well as quantitative tobacco exposure (PY), was missing. Many studies were too small to detect small differences between particular patient populations. Finally, the studies varied in duration of follow-up, analysis methods, and endpoints.

## 10. Conclusions

Tobacco smoking is probably the strongest modifiable factor affecting the outcome of cancer treatment. To better characterize its effect on RT outcomes, it is advisable to document a thorough smoking history in routine clinical practice and prospective clinical studies. Patients should be aware of smoking hazards and be advised when they and their families are being counseled. Healthcare professionals should use the cancer diagnosis and treatment as a teachable moment and recommend immediate smoking cessation [69]. Wherever possible, cancer patients should undergo an intensive smoking cessation program, including behavioral and pharmacologic therapy.

## Figures and Tables

**Table 1:** Summary of analyzed studies.

First Author, Year (Ref)	*N* Patients	Stage	Treatment	Current Smokers	Past Smokers	Never Smokers	Major Findings	Analysis
Non-small cell lung cancer								
Tsao 2006 [8]	497	III–IV	CHRT	232	215	30	One-year OS in never, former, and current smokers was 69%, 67%, and 67%, respectively (*p* = 0.5)	MVA
Nguyen 2010 [9]	152	I–III	RT, Surgery	49	98	5	Higher risk of LRC in smokers vs. non-smokers (HR 3.6; *p* = 0.0006)	MVA
Fisher-Valuck 2013 [10]	62	I	SBRT	18	34	10	HR for death in smokers vs. never-smokers 1.31; *p* = 0.341; treatment-related toxicity 14.5% and 0%, respectively	UVA
Roach 2015 [11]	119	Early	SBRT	87	32	0	Improved two-year OS in quitters compared to continued smoking (78% vs. 69%; *p* = 0.014)	MVA
Miller 2019 [12]	226	I–IV	SBRT	55	156	15	HR for distant failure in current vs. never-smokers 0.76 (0.24–2.36) and for former vs. never-smokers 0.87 (0.31–2.46)	UVA
Fox 2003 [13]	237	I–IV	RT, CHRT	30	188	19	Two-year OS 41% in smokers vs. 56% in non-smokers (*p* = 0.01)	MVA
Sheikh ^d^ 2021 [14]	517	I–III	RT, CHT, CHRT	297	220	0	Impaired LRC in current vs. former smokers (HR 1.74; *p* = 0.029)	MVA
Rades 2008 [15]	181	I–III	RT, CHRT, Surgery	74	0	107 *	Higher risk of LRC in smokers vs. non-smokers (RR 1.74; *p* = 0.029)	MVA
Hernando 2001 [16]	201	I–IV	RT, CHRT	68	0	133 *	RP toxicity 21% in smokers vs. 37% in non-smokers (*p* = 0.05)	UVA
Jin 2009 [17]	576	I–IV	RT, CHRT, CHT	156	374	46	Higher one-year incidence of grade ≥ 3 RP toxicity in never vs. former or current smokers (37% vs. 14%; *p* = 0.001)	UVA
Menoux 2020 [18]	83	I	RT	19	67	2	Lower RP toxicity in active smokers vs. non-smokers (*p* = 0.02)	UVA
Monson 1998 [19]	83	I–IV	RT, Surgery	78 *	0	5	RP toxicity 23% in smokers vs. 0% in non-smokers (*p* = 0.01)	UVA
Sarihan 2005 [20]	181	I–IV	RT, CHRT	NR	NR	NR	Risk of infections during treatment associated with tobacco exposure (*p* = 0.001)	MVA
Kawaguchi 2006 [21]	62	III–III	CHRT	16	19	10	Higher risk of SPC in continued smokers vs. general population (RR 5.2; 1.6–11.7)	UVA
Small cell lung cancer								
Johnston 1980 [22]	112	Limited, Extensive	CHRT, CHT	57	54	1	Median OS 47, 52, and 70 weeks in smokers, quitters at diagnosis, and quitters before diagnosis, respectively (*p* = 0.04)	UVA
Videtic 2003 [23]	215	Limited	CHRT	79	0	107 *	Median OS in smokers vs. non-smokers (18 vs. 13.6 months; *p* = 0.002)	MVA
Tucker 1997 [24]	611	Limited, extensive	CHRT	214	325	13	RR of second lung cancer 9.1 and 21 in quitters and continued smokers compared to general population, respectively	MVA
Kawahara 1998 [25]	70	Limited	CHRT, CHT	33	31	5	HR for SPC in current vs. non-smokers 4.3 (*p* = 0.03)	MVA
Head and neck cancer								
Browman 1993 [26]	115	III–IV	RT	53	58	4	Impaired OS in smokers vs. non-smokers (HR 2.5; *p* = 0.002)	MVA
Browman 2002 [27]	148	III–IV	RT	113	0	35 *	HR for death in smokers at recruitment vs. non-smokers or very light smokers 1.17 (*p* = 0.07)	MVA
Kawakita ^b^ 2011 [28]	107	I–IV	RT, CHRT, Surgery	64	0	43 *	HR for OS 5.31 (*p* = 0.039) in moderate smokers, 5.35 (*p* = 0.084) in heavy smokers, and 8.42 (*p* = 0.016) in non-smokers, compared to light smokers	MVA
Hoff 2012 [29]	232	I–V	RT	162	58	12	Impaired OS in smokers vs. non-smokers (HR 1.81; *p* = 0.03); impaired LRC in smokers vs. non-smokers (HR 1.96; *p* = 0.02)	MVA
Fortin 2009 [30]	1871	NA	RT, CHRT, Surgery	951	755	165	Impaired LCR in current vs. former smokers (HR 1.5; *p* = 0.0001); impaired OS in current and former vs. never smokers (HR 1.75 and 1.33, respectively; *p* = 0.000001)	MVA
Gillison 2012 [31]	506	III–IV	RT, CHRT	160	218	96	HR for OS in smokers during treatment vs. non-smokers 2.18 (*p* = 0.001); HR for PFS in smokers during treatment vs. non-smokers 2.04 (*p* = 0.001)	MVA
Lassen ^d^ 2019 [32]	1875	NR	RT, CHRT	425	853	567	HR for OS, PFS and DFS in smokers vs. non-smokers 2.06 (1.49–2.84), 1.73 (1.18–2.53) and 1.79 (1.35–2.36), respectively	MVA
Smith 2019 [33]	6332	NR	RT	455	872	0	RR for OS and LRC in current smokers vs. ceased smokers 1.85 and 2.24 (*p* = 0.0001, *p* = 0.0005, respectively)	SR
Chen 2019 [34]	63	I–IV	CHRT	22	0	41 *	Three-year PFS 34% in current vs. 61% in former smokers (HR 0.4; *p* = 0.05)	MVA
Egestad 2014 [35]	65	T1–4, N0–2	CHRT, Surgery	13	36	16	Increased fatigue (*p* = 0.027) and pain (*p* = 0.009), more speech disturbances (*p* = 0.017), affected mouth opening (0.049) and poorer cognitive function (0.041) in smokers vs. non-smokers	MVA
Jensen 2007 [36]	114	I–IV	RT, Surgery	52	48	14	Decreased cognitive function, increased nausea/vomiting, dyspnoea, diarrhea, and weight loss in smokers vs. non-smokers (*p* = 0.05)	UVA
Silveira 2015 [37]	110	T0–4, N0–3	RT, CHRT	44	0	66 *	Worse burden (*p* = 0.003), mental health (*p* = 0.03), and fatigue (*p* = 0.028) in continued smokers vs. non-smokers	UVA
Guo ^c^ 2014 [38]	400	III–IV	CHRT	159	34	207	Impaired LRFFS in ever-smokers vs. never-smokers (HR 2.2; *p* = 0.002)	MVA
Sun ^c^ 2021 [39]	23,325	I–IVa	RT, CHRT	12,944 *	0	10,381	Five-year OS 69% in current vs. 76% in former smokers vs. 80% in never smokers (*p* = 0.003)	MVA
Al-Mamagani ^a^ 2013 [40]	549	T1a	RT	421	0	137 *	Impaired LC and OS in current vs. former smokers (*p* = 0.001 for both comparisons, increased risk of SPC (*p* = 0.003)	MVA
Johannes ^a^ 1998 [41]	383	T1N0M0	CHRT	97	180	0	Ten-year complication rate 28% vs. 14% in smokers vs. quitters before RT (*p* = 0.03)	MVA
Porock 2004 [42]	53	NR	RT	16	33	0	More frequent oral mucositis in smokers (*p* = 0.03), mucositis severity associated with higher tobacco exposure (*p* = 0.008)	UVA
Rugg 1990 [43]	41	III–IV	RT	8	25	8	More skin reactions and oral mucositis in current vs. former and never-smokers	UVA
Khuri 2006 [44]	1190	I/II	RT, Surgery	456	574	160	Increased mortality for current vs. never smokers (HR 2.51; 1.54–4.10) and for current vs. former smokers (HR 1.60; 1.23–2.07)	MVA
Breast cancer								
DiMarzio 2018 [45]	14,106	I–III	RT	988	2930	6218	Increased mortality in ever vs. never-smokers (HR 1.25; 1.06–1.47)	MVA
Obedian 2020 [46]	2416	I–IV	RT, CHRT	210	190	448	15-year risk of SPC 20% in smokers vs. 16% in non-smokers (*p* = 0.07)	UVA
Sharp 2013 [47]	390	NR	RT	32	352		More severe acute radiation skin reactions in current smokers vs. non-smokers (HR 2.5; *p* = 0.031)	MVA
Hooning 2007 [48]	7425	I–IIIa	RT, CHRT, Surgery	743 *	2376 *	49	Increased risk of myocardial infarction in smokers during RT vs. non-smokers (HR 3.04; *p* = 0.039)	MVA
Wong 2020 [49]	NR	NR	RT	NR	NR	NR	Increased prevalence of failure rate of capsular contracture reconstruction, and other major complications in smokers vs. non-smokers	SR
Prostate cancer								
Taira 2011 [50]	1656	T1b–T3a	RT	258	764	631	Increased mortality in current vs. never-smokers (HR 2.9; *p* = 0.001)	MVA
Merrick 2006 [51]	938	T1b–T3a	RT	161	491	286	Increased mortality in current vs. never-smokers (RR 4.3; *p* = 0.001)	MVA
Pantarotto 2006 [52]	416	T1–4	RT	70	226	120	HR for OS in current vs. never-smokers 1.72 (*p* = 0.08); HR for distant control 5.24 (*p* = 0.003)	MVA
Pickles 2004 [53]	601	T1–4	RT	88	329	184	Six-year mortality 26% in smokers vs. 11% in non-smokers (*p* = 0.009)	MVA
Alsadius 2011 [54]	985	Localized	RT	82	401	353	Higher prevalence of abdominal cramps, defecation urgency, sensation of bowel not completely emptied after defecation, sudden emptying of bowels into clothing without forewarning in current vs. non-smokers (HR 3.5, 1.5, 2.1 and 4.7, respectively; *p* = 0.004, <0.001, 0.003 and 0.003, respectively)	MVA
Boorjian 2007 [55]	9780	T1–T4	RT, Surgery	742	0	6718 *	Increased risk of secondary bladder cancer in smokers during RT vs. non-smokers (HR 3.65; 1.45–9.16)	MVA
Foerster 2018 [56]	22,549	NR	RT, Surgery	4202	0	18,347 *	HR for biochemical recurrence in current and former-smokers receiving RT 1.50 (1.20–1.88) and 1.10 (0.94–1.28), respectively	SR
Cervical cancer								
Mayadev 2018 [57]	96	I–III	RT, CHRT	45 *	0	51	HR for OS in smokers (1–20 PYs) vs. non-smokers 4.68 (*p* = 0.047)	MVA
Eifel 2002 [58]	3489	I–II	RT	1173	123	2083	Higher incidence of small bowel, rectal and bladder complications in smokers ≥ 1 pack vs. <1 pack per day (HR 3.25, 2.20 and 1.81, respectively; *p* = 0.0005, <0.0005 and 0.006, respectively)	MVA
Fyles 2002 [59]	115	I–III	RT	34	0	66 *	Smoking status not associated with PFS and LC	MVA
Oesophageal cancer								
Zou 2019 [60]	497	II–IV	RT, CHRT	265	43	171	Higher mortality in former and current vs. never-smokers HR 1.57; *p* = 0.01)	MVA
Anal cancer								
Lerman 2020 [61]	171	T1–4, N0/+	RT, CHRT	28	58	85	Impaired PFS in smokers vs. never-smokers (HR 2.85; *p* = 0.013)	MVA
Hodgkin lymphoma								
Leeuwen 1995 [62]	112	I–IV	RT, CHT, CHRT	78 *	0	34 *	Higher risk of secondary lung cancer in >10 PY vs. <10 PY smokers (HR 6.2; *p* = 0.03)	MVA
Mixed malignancies								
Peppone 2011 [63]	947	NR	RT, CHRT	85	17	632	Higher mean total symptom burden during treatment in smokers vs. non-smokers (46% vs. 41%, respectively; *p* = 0.048)	MVA
Wells 2004 [64]	357	Localized	RT	82	126	148	Higher RTOG scores (*p* = 0.0001), erythema meter mean (*p* = 0.009), and mean diary score (*p* = 0.016) in smokers vs. non-smokers	MVA

Legend: *—Exact tobacco status not well described; not direct active smokers classified as non-smoker; ^a^ glottic larynx cancer only; ^b^ oral cavity squamous cell cancer patients only; ^c^ nasopharyngeal cancer patients only; ^d^ conference abstract; CHRT—chemoradiotherapy; CHT—chemotherapy; CR—complete response; DFS—disease-free survival; HR—hazard ratio; LC—lung cancer; LRC—locoregional control; LRFFS—locoregional recurrence failure-free survival; MVA—multivariate analysis; NR—not reported; OS—overall survival; PC—prostate cancer; PFS—progression-free survival; PR—partial response; PY—pack-years; RTOG—Radiation Therapy Oncology Group; QoL—quality of life; RP—radiation pneumonitis; RR—relative risk; RT—radiotherapy; RTOG—Radiation Therapy Oncology Group; SBRT—stereotactic body radiation therapy; SPC—second primary cancer; SR—systematic review; UVA—univariate analysis.

**Table 2:** Impact of tobacco smoking on overall survival.

First Author, Year (Ref)	Malignancy	Outcome
Tsao 2006 [8]	NSCLC	One-year OS in never, former, and current smokers 69%, 67%, and 67%, respectively (NS)
Nguyen 2010 [9]	NSCLC	Five-year OS in smokers and non-smokers 20% and 34%, respectively (NS)
Fisher-Valuck 2013 [10]	NSCLC	HR for death in smokers vs. never-smokers 1.31 (NS)
Roach 2015 [11]	NSCLC	Two-year OS in quitters and continued smokers 78% and 69%, respectively (*p* = 0.014)
Fox 2003 [13]	NSCLC	Two-year OS in smokers and non-smokers 41% and 56%, respectively (*p* = 0.01)
Sheikh 2021 [14]	NSCLC	Five-year OS in current and ceased smokers 49% and 61%, respectively (*p* = 0.001)
Rades 2008 [15]	NSCLC	Two-year OS 25% in smokers and non-smokers 25% and 39%, respectively (NS)
Johnston 1980 [22]	SCLC	Median OS in smokers, quitters at diagnosis, and quitters before diagnosis 47, 52, and 70 weeks, respectively (*p* < 0.04)
Videtic 2003 [23]	SCLC	Median OS in smokers and non-smokers 18 and 13.6 months, respectively (*p* = 0.002)
Browman 1993 [26]	HNC	HR for OS in smokers and non-smokers 2.5 (*p* = 0.002)
Browman 2002 [27]	HNC	HR for OS in smokers at presentation vs. non-smokers or very light smokers 1.17 (NS)
Kawakita 2011 [28]	HNC	HR for OS in moderate smokers, heavy smokers and non-smokers, 5.31 (*p* = 0.039), 5.35 (*p* = 0.084) and 8.42 (*p* = 0.016), respectively, compared to light smokers
Hoff 2012 [29]	HNC	HR for OS in smokers vs. non-smokers HR 1.81 (*p* = 0.03)
Fortin 2009 [30]	HNC	HR for OS in current and former vs. never-smokers 1.75 and 1.33, respectively (*p* = 0.000001)
Gillison 2012 [31]	HNC	HR for OS in smokers during treatment vs. non-smokers 2.18 (*p* < 0.001)
Lassen 2019 [32]	HNC	HR for OS in smokers vs. non-smokers 2.06 (1.49–2.84)
Smith 2019 [33]	HNC	RR for OS in current vs. ceased smokers 1.85 (*p* < 0.0001)
Chen 2019 [34]	HNC	Three-year OS in current and ceased smokers 71% and 67%, respectively (*p* = 0.42)
Guo 2014 [38]	HNC	Five-year OS in light, short-term smokers, light, long-term smokers, heavy, short-term smokers and heavy, long-term smokers 62%, 78%, 74%, and 63%, respectively (NS)
Sun ^c^ 2021 [39]	HNC	Five-year OS in current, former, and never-smokers 69%, 76%, and 80%, respectively (*p* < 0.003)
Al-Mamagani 2013 [40]	HNC	Ten-year OS in current and ceased smokers 36% and 70%, respectively (*p* < 0.001)
Khuri 2006 [44]	HNC	HR for OS in current or former vs. never-smokers (2.53 and 1.53, respectively (*p* < 0.0001)
DiMarzio 2018 [45]	BC	HR for OS in ever vs. never-smokers 1.25 (*p* < 0.05)
Taira 2011 [50]	PC	HR for OS in current vs. never-smokers 2.9 (*p* < 0.001)
Merrick 2006 [51]	PC	RR for OS in current vs. never-smokers 4.3 (*p* < 0.001)
Pantarotto 2006 [52]	PC	HR for OS in current vs. never-smokers 1.72 (NS)
Pickles 2004 [53]	PC	Six-year mortality in smokers and non-smokers 26% and 11%, respectively (*p* = 0.009)
Foerster 2018 [56]	PC	HR for cancer-specific mortality in current or former vs. never smokers 2.03 (NS), and 1.66 (NS), respectively
Mayadev 2018 [57]	CC	HR for OS in smokers (1–20 PYs) vs. non-smokers 4.68 (*p* = 0.047)
Zou 2019 [60]	OC	HR for OS in former and current vs. never-smokers 1.57 (*p* = 0.01)
Lerman 2020 [61]	AC	Five-year OS in smokers and non-smokers 30% and 33%, respectively (*p* = 0.03)

Legend: AC—anal cancer; BC—breast cancer; CC—cervical cancer; HNC—head and neck cancer; HR—hazard ratio; NSCLC—non-small cell lung cancer; NS—non-significant; OC—esophageal cancer; OS—overall survival; PC—prostate cancer; PY—pack-years; SCLC—small cell lung cancer; ^c^ nasopharyngeal cancer patients only.

**Table 3:** Impact of tobacco smoking on locoregional and distant control.

First Author, Year (Ref)	Malignancy	Outcome
Nguyen 2010 [9]	NSCLC	HR for LRC in smokers vs. non-smokers 3.6 (*p* = 0.0006)
Miller 2019 [12]	NSCLC	HR for distant failure in current vs. never-smokers 0.76 (0.24–2.36) and for former vs. never-smokers 0.87 (0.31–2.46)
Sheikh ^d^ 2021 [14]	NSCLC	HR for LRC in current vs. former-smokers 1.74 (*p* = 0.029)
Rades 2008 [15]	NSCLC	RR for LRC in smokers vs. non-smokers 1.74 (*p* = 0.029)
Kawakita 2011 [28]	HNC	No significant association between relapse and smoking status was observed (*p* = 0.370)
Hoff 2012 [29]	HNC	HR for LRC in smokers vs. non-smokers 1.96 (*p* = 0.02)
Fortin 2009 [30]	HNC	HR for LCR in current vs. former-smokers 1.5 (*p* = 0.0001)
Gillison 2012 [31]	HNC	HR for PFS in smokers during treatment vs. non-smokers 2.04 (*p* = 0.001)
Lassen 2019 [32]	HNC	HR for PFS and DFS in smokers vs. non-smokers 1.73 (1.18–2.53) and 1.79 (1.35–2.36), respectively
Smith 2019 [33]	HNC	RR for LRC in current vs. ceased smokers 2.24 (*p* = 0.0005)
Chen 2019 [34]	HNC	Three-year PFS in current and former-smokers 34% and 61%, respectively (*p* = 0.05)
Guo 2014 [38]	HNC	HR for LRFFS in ever-smokers vs. never-smokers 2.2 (*p* = 0.002)
Sun ^c^ 2021 [39]	HNC	Higher locoregional recurrence risk in current vs. never-smokers (*p* = 0.027); higher metastasis risk in former and current vs. never-smokers (*p* = 0.031 and 0.019, respectively)
Al-Mamagani 2013 [40]	HNC	OR for LC in current vs. ceased smokers 3.8 (*p* = 0.001)
Khuri 2006 [44]	HNC	HR for DFS in current or former vs. never-smokers 1.68 and 1.26, respectively (*p* = 0.011)
Pantarotto 2006 [52]	PC	HR for distant control in current vs. never-smokers 5.24 (*p* = 0.003)
Foerster 2018 [56]	PC	HR for biochemical recurrence in current or former vs. never smokers 1.50 (1.20–1.88) and 1.10 (0.94–1.28), respectively
Mayadev 2018 [57]	CC	Impaired two-year pelvic control and DFS in heavy smokers (>21 PY) vs. non-smokers (*p* = 0.004 and 0.011, respectively)
Fyles 2002 [59]	CC	Smoking status not associated with PFS and LC
Lerman 2020 [61]	AC	HR for PFS in smokers vs. never-smokers 2.85 (*p* = 0.013)

Legend: AC—anal cancer; CC—cervical cancer; DFS—disease-free survival; HNC—head and neck cancer; HR—hazard ratio; LRC—locoregional control; LRFFS—locoregional recurrence failure-free survival; NSCLC- non-small cell lung cancer; NS—non-significant; OR—odds ratio; PC—prostate cancer; PFS—progression-free survival; PY—pack-years; RR—relative risk; ^c^ nasopharyngeal cancer patients only; ^d^ conference abstract.

**Table 4:** Impact of tobacco smoking on risk of developing second primary cancer.

First Author, Year (Ref)	Malignancy	Outcome
Kawaguchi 2006 [21]	NSCLC	RR for SPC in continued smokers vs. general population 5.2 (1.6–11.7)
Tucker 1997 [24]	SCLC	RR for secondary lung cancer 9.1 and 21 in ceased and continued smokers compared to general population, respectively
Kawahara 1998 [25]	SCLC	HR for SPC in current smokers vs. non-smokers 4.3 (*p* = 0.03)
Kawakita 2011 [28]	HNC	No significant association between SPC and smoking status
Al-Mamagani 2013 [40]	HNC	Ten-year SPC risk in current and former-smokers 21% and 12%, respectively (*p* = 0.003)
Khuri 2006 [44]	HNC	HR for SPC-free survival in current or former vs. never-smokers 1.64 (1.09–2.48) and 1.20 (0.80–1.82), respectively
DiMarzio 2018 [45]	BC	Higher risk of SPC in ever- vs. never-smokers (*p* = 0.04).
Obedian 2020 [46]	BC	15-year SPC risk in smokers and non-smokers 20% and 16%, respectively (NS)
Boorjian 2007 [55]	PC	HR for risk of secondary bladder cancer in smokers vs. non-smokers 3.65 (1.45–9.16)
Leeuwen 1995 [62]	HL	HR for risk of secondary lung cancer in >10 PY vs. <10 PY smokers 6.2 (*p* = 0.03)

Legend: BC—breast cancer; HNC—head and neck cancer; HR—hazard ratio; NSCLC—non-small cell lung cancer; NS—non-significant; PC—prostate cancer; PY—pack-years; RR—relative risk; SCLC—small cell lung cancer; SPC—second primary cancer.

**Table 5:** Impact of tobacco smoking on treatment toxicity and complications.

First author, Year (Ref)	Malignancy	Outcome
Fisher-Valuck 2013 [10]	NSCLC	Treatment-related toxicity 14.5% in current or past smokers and 0% in never smokers
Hernando 2001 [16]	NSCLC	RP occurrence in smokers and non-smokers 21% and 37%, respectively (*p* = 0.05)
Jin 2009 [17]	NSCLC	One-year incidence of grade ≥3 RP in never vs. former or current smokers 37% and 14%, respectively (*p* = 0.001)
Menoux 2020 [18]	NSCLC	Lower RP occurrence in active smokers vs. non-smokers (*p* = 0.02)
Monson 1998 [19]	NSCLC	RP occurrence in smokers and non-smokers 23% and 0%, respectively (*p* = 0.01)
Sarihan 2005 [20]	NSCLC	Risk of infections during treatment associated with tobacco exposure (*p* = 0.001)
Egestad 2014 [35]	HNC	Increased fatigue (*p* = 0.027) and pain (*p* = 0.009), more speech disturbances (*p* = 0.017), affected mouth opening (0.049) and poorer cognitive function (0.041) in smokers vs. non-smokers
Jensen 2007 [36]	HNC	Decreased cognitive function, increased nausea/vomiting, dyspnoea, diarrhea, and weight loss in smokers vs. non-smokers (*p* = 0.05)
Silveira 2015 [37]	HNC	Worse burden (*p* = 0.003), mental health (*p* = 0.03), and fatigue (*p* = 0.028) in continued smokers vs. non-smokers
Al-Mamagani 2013 [40]	HNC	Smoking quitters showed better voice quality during two-year follow-up (*p* = 0.001 for all time points)
Johannes 1998 [41]	HNC	Ten-year complication rate 28% vs. 14% in smokers vs. quitters before RT (*p* = 0.03)
Porock 2004 [42]	HNC	More frequent oral mucositis in smokers (*p* = 0.03), mucositis severity associated with higher tobacco exposure (*p* = 0.008)
Rugg 1990 [43]	HNC	More skin reactions and oral mucositis in current vs. former and never-smokers
Sharp 2013 [47]	BC	HR for severe acute radiation skin reactions in current smokers vs. non-smokers HR 2.5 (*p* = 0.031)
Hooning 2007 [48]	BC	HR for myocardial infarction in smokers during RT vs. non-smokers 3.04 (*p* = 0.039)
Wong 2020 [49]	BC	Increased prevalence of failure rate of capsular contracture reconstruction, and other major complications in smokers vs. non-smokers
Alsadius 2011 [54]	PC	HR for abdominal cramps, defecation urgency, sensation of bowel not completely emptied after defecation, sudden emptying of bowels into clothing without forewarning in current vs. non-smokers (3.5 (*p* = 0.004), 1.5 (<0.001), 2.1 (0.003) and 4.7 (0.003), respectively
Eifel 2002 [58]	CC	HR for small bowel, rectal and bladder complications in smokers ≥1 pack vs. <1 pack per day 3.25 (*p* = 0.0005), 2.20 (<0.0005) and 1.81 (0.006), respectively

Legend: BC—breast cancer; CC—cervical cancer; HNC—head and neck cancer; HR—hazard ratio; NSCLC—non-small cell lung cancer; PC—prostate cancer; RP—radiation pneumonitis; RR—relative risk; RT—radiotherapy.
